# Effectiveness and safety of traditional Chinese medicine in treating acquired immune deficiency syndrome: 2004–2014

**DOI:** 10.1186/s40249-015-0093-6

**Published:** 2015-12-23

**Authors:** Zhi-Bin Liu, Ji-Ping Yang, Li-Ran Xu

**Affiliations:** Department of Acquired Immune Deficiency Syndrome Treatment and Research Center, the First Affiliated Hospital of Henan University of Traditional Chinese Medicine, 19 Ren Min Road, Zhengzhou, 450000 Henan China; Discipline of Internal Chinese Medicine, the First Affiliated Hospital of Henan University of Traditional Chinese Medicine, Zhengzhou, Henan China; Department of Cellular Immunology Laboratory, Key Laboratory of Viral Diseases Prevention and Treatment of Traditional Chinese Medicine of Henan Province, Zhengzhou, 450000 Henan China

**Keywords:** HIV/AIDS, Traditional Chinese medicine, Antiretroviral therapy, Herbal medicine, Quality of life, Survival, Immune reconstitution

## Abstract

Substantial progress has been made in China in using traditional Chinese medicine (TCM) to treat acquired immune deficiency syndrome (AIDS). Our objective was to review the latest developments in TCM treatment of AIDS in China between 2004 and 2014. We reviewed the content of original articles investigating the efficacy and safety of TCM for treating AIDS published in Chinese and English language journals. Relevant references from 2004 to 2014 were found using PubMed and the China National Knowledge Infrastructure Database. We found that TCM has been widely used for treating AIDS and its complications in China. The number of TCM studies has increased, which indicates efficacy and safety. Measures of efficacy in the reviewed articles included the alleviation of human immunodeficiency virus (HIV)-related signs and symptoms, improvements in quality of life, improvements in long-term survival, counteraction of the adverse side effects of antiviral drugs, promotion of immune reconstitution, and improvement of laboratory results. In sum, the literature indicates that TCM is safe. TCM plays an important role in the treatment of AIDS. Some studies have attempted to measure the efficacy and safety of TCM for treating AIDS, but more evidence is needed. Therefore, more research on this topic is required in the future.

**Electronic supplementary material:**

The online version of this article (doi:10.1186/s40249-015-0093-6) contains supplementary material, which is available to authorized users.

## Multilingual abstracts

Please see Additional file 1 for translations of the abstract into the six official working languages of the United Nations.

## Introduction

Acquired immune deficiency syndrome (AIDS) is a type of communicable disease caused by human immunodeficiency virus (HIV) infection [1]. AIDS was first recognized in the early 1980s. By the end of 2014, 36.9 million [34.3 million-41.4 million] people were living with HIV, and 1.2 million [980,000–1.6 million] people died from AIDS-related illnesses [2]. This global tragedy is likely to continue into the foreseeable future. The prevention and control of AIDS, and the development of adequate therapies, is challenging. It will be a herculean and unpredictable task to find an effective cure for the disease.

Fortunately, methods such as community-based interventions have improved knowledge, attitudes, and practice outcomes for AIDS [3, 4]. Mortality and morbidity have been reduced since combination antiretroviral therapy (cART) was introduced in 1996 [5]; cART has led to a notable increase in survival and quality of life (QOL) and has substantially improved the health status of HIV infected patients [6, 7].

At present, cART is the most effective treatment strategy for AIDS, and has the advantage of immunohematologic and virologic outcomes. However, the lifelong medication involved in cART is often limited by drug toxicity, poor adherence to therapy, and drug resistance [8]. It is therefore necessary to find new drugs or treatments, such as complementary and alternative medicine (CAM), to treat AIDS and its complications to achieve better resistance, drug adherence, and treatment tolerability.

CAM has been used worldwide to treat AIDS and its complications, and includes traditional Chinese medicine (TCM), acupuncture and moxibustion, and qigong. Some clinical research has demonstrated that TCM is effective in treating AIDS [9]. Since 2004, there have been several efforts to explore the use of TCM in AIDS treatment. For example, a national trial has been conducted in the five major provinces of China to investigate the effect of TCM on AIDS, and related research has been funded by the National Special Science and Technology Program on Major Infectious Diseases [10]. Additionally, some new modalities for treating AIDS with TCM have been developed [11].

TCM and Western medicine (WM) use different strategies to treat AIDS. WM theory is mainly based on physiological structures or anatomy, and conceives the therapy goal as the elimination or removal of the pathogen. TCM emphasizes the holistic nature of the body and the subjective experiences of patients and practitioners. TCM disorders arise from external pathological factors or an imbalance of yin and yang, and the therapy goals are the harmonization of internal function and external natural environmental changes. Thus, the main goals of WM, especially cART, are to eliminate, diminish, or suppress the viral load levels in the body and to improve the immune system. CD4 + T cell counts and HIV loads are gold standard evaluation criteria for AIDS therapy as recommended by the World Health Organization (WHO). In contrast, TCM places less emphasis on objective laboratory examination results, such as CD4 + T cell counts and levels of HIV loads, and focuses more on AIDS patients’ clinical complaints, such as abnormal symptoms and signs, lower QOL, complications, opportunistic infections, and cART side effects [12, 13].

In this paper, we summarize the findings of studies on the efficacy and safety of TCM for treating HIV/AIDS in the 10 years from 2004 to 2014.

## Alleviating HIV-related signs and symptoms

The signs and symptoms of AIDS result from conditions that do not normally develop in individuals with healthy immune systems. Patients infected with HIV often complain of many signs and symptoms (such as diarrhea, fever, fatigue, insomnia, weakness, and weight loss) [14], especially when using cART to halt disease progression and prolong the lifespan. These symptoms may lower patients’ QOL, reduce therapy compliance, and even lead to the failure of antiviral treatment.

One of the advantages of TCM is that it can alleviate HIV-related signs and symptoms. At present, there are two main TCM approaches for this. One targets a single symptom and uses a corresponding fixed herbal preparation or strategy to relieve or ease it. The other aims to prevent the occurrence of self-reported symptoms by using fixed-combination herbal preparations combined with syndrome differentiation.

HIV/AIDS patients often report diarrhea; this has many potential causes, including intestinal parasites. Xie Li Kang capsule is a fixed-combination Chinese herbal hospital preparation with the TCM properties of warming the mid-section, invigorating the spleen, producing an insecticidal action, and relieving diarrhea with astringents. Its main ingredients include *Galla chinensis*, *Myristica eragrans*, *Cortex dictamni*, and garlic. In a randomized, double-blinded, double-dummy, controlled clinical trial ending in 2011 to test Xie Li Kang capsule on HIV-related diarrhea [15], the primary efficacy parameters were stool weight, abnormal stool frequency, and diarrhea questionnaire scores. The results showed that participants taking Xie Li Kang capsule experienced significantly reduced stool weight and diarrhea questionnaire scores compared with the controlled group. There were no significant differences in stool frequency.

Yi Ai Kang capsule is a Chinese hospital herbal preparation that strengthens the spleen, dries damp, invigorates vital energy, and nourishes blood. Its main ingredients include *Astragalus membranaceus*, ginseng, *Ligusticum chuanxiong*, Radix *Paeoniae alba*, and *Scutellaria baicalensis*. It has been used to treat HIV/AIDS in Henan Province, China, and more than 4,500 people with the disease take this preparation every day [11]. One cohort study evaluated the effects of Yi Ai Kang capsule on 379 asymptomatic HIV patients not on cART [16]. The results suggested that the treatment was effective in reducing fatigue, shortness of breath, and spontaneous perspiration.

Aining granule tonifies qi, nourishes yin, and invigorates the spleen; it consists of Radix *Astragali mongolici*, *Fructus lycii*, *Panax notoginseng*, *Poria cocos*, and Radix *et Rhizoma glycyrrhizae*. One randomized, double-blinded, placebo-controlled clinical trial tested Aining granule plus cART on 100 HIV/AIDS cases [17]. Although CD4 + T cell counts decreased in both groups after 11 months, CD4 + T counts declined less in the Aining group than in the control group. The treatment group showed significant improvement of symptoms such as fatigue, anorexia, nausea, diarrhea, and skin rash. However, there was no significant difference between the two groups in viral load after treatment.

Classic clinical TCM treatment based on syndrome differentiation has been used to treat HIV-related signs and symptoms. A multicenter, stratified, randomized, parallel-group trial of 164 subjects evaluated the effects of TCM on clinical symptoms of AIDS with pulmonary inflammation [18]. A randomized, stratified sample was divided into two regimens: the TCM–WM group (TCM treatment based on syndrome differentiation plus WM routine treatment) and the WM group (WM routine treatment). The incidence of TCM symptoms at different time points was analyzed. The findings suggested that integrated TCM–WM treatment helps to alleviate the clinical symptoms of AIDS with pulmonary inflammation.

In summary, Chinese medicine professionals (CMPs) have used TCM to alleviate HIV-related signs and symptoms, and many observations and trials have been reported. However, the quality of this research is not high; therefore, the effectiveness of TCM for HIV-related signs and symptoms is still uncertain.

## Improving patients’ QOL

QOL has been defined as “an individual’s perception of their position in life in the context of the culture and value systems in which they live and in relation to their goals, expectations, standards and concerns” [19]. QOL is a multidimensional concept related to physical, psychological, and social functioning. As the QOL of HIV/AIDS patients is substantially lower than the general population, it has been a focus of both TCM and WM research on HIV/AIDS. CMPs believe that TCM is a convenient, personalized, inexpensive, and effective healthcare method and may therefore improve the QOL of HIV/AIDS patients [20]. QOL is often used as an assessment indicator for comprehensive TCM AIDS treatments.

Treatment with Chinese herbal medicine based on TCM syndrome differentiation might improve QOL for people with AIDS. A prospective TCM study into QOL in asymptomatic HIV-infected persons was carried out from June 2009 to December 2010 in China [21]. This was a randomized, double-blinded, parallel, placebo-controlled study on 1,200 patients in the asymptomatic period of HIV infection for 18 months. The WHO QOL-HIV scale was used to assess QOL at the time points 0 days, 6 months, 12 months, and 18 months. The QOL of the treatment group increased more than that of the control group, which suggests that treatment based on TCM syndrome differentiation can significantly improve patients’ QOL.

Fixed-combination Chinese herbal hospital preparations may also improve QOL in people with AIDS. Aikang capsule contains 21 Chinese herbs, including *Panax quinquefolius*, *Dioscorea opposita*, *Astragalus membranaceus*, *Poria cocos*, *Atractylodes macrocephala*, Radix *Rehmanniae preparata*, *Angelica sinensis*, *Colla corii asini*, Radix *Paeoniae alba*, and *Ophiopogon japonicus*; this capsule tonifies qi and activates blood circulation, strengthens the spleen, tonifies the lung, dries damp, expels wind, and removes dampness. In a randomized, single-blind, parallel, placebo-controlled study on 102 HIV/AIDS patients not taking cART [22], the Nottingham Health Profile was used at 0 days, 3 months, 6 months, and 9 months during follow-up. The results showed that Aikang capsule can mitigate symptoms of HIV/AIDS patients, such as pain and reduced physical activity, by replenishing energy and nurturing general well-being to improve patients’ QOL.

To summarize, QOL has been used as an indicator to assess TCM treatment for HIV/AIDS in China and other countries. However, research to date has been limited and more evidence is needed to confirm TCM’s effectiveness.

## Benefits for long-term survival of AIDS patients

AIDS is still a fatal disease with limited therapeutic options. Among infectious diseases, HIV/AIDS has become the leading cause of death in many countries. Survival is an important prognostic index and can be used to express the serious lethality of the disease and to evaluate long-term curative effects [23]. Recently, some CMPs have reported results from studies of the survival of HIV/AIDS patients treated with TCM.

In a prospective cohort study, 165 of 385 AIDS patients used a 16-herb Chinese medicine anonymity formula for 14 days to 9 months [24]. The 8-year survival rate was 87 % for the treatment group and 34 % for the non-treatment group. There were no deaths in patients who used Chinese herbal medicine for 6–9 months, and none of the survivors experienced any recurrence of AIDS or any severe adverse events. This suggests that Chinese herbal medicine might improve the long-term survival of AIDS patients.

In a 6-year retrospective cohort study [25], 1,666 people living with HIV in Henan province of China were treated with comprehensive TCM intervention from 2004 to 2010 with 102,591 person-months of follow-up. Participants received a fixed preparation of Yi Ai Kang capsules, plus additional TCM treatment according to each individual patient’s symptoms using syndrome differentiation. Overall, 312 (18.7 %) patients died over the 6-year study period. The total mortality rate over the study period was 3.6 per 100 person-years, which is lower than the worldwide rate [26]. The authors reported that TCM increased the survival and lengthened the lifespan of people living with HIV. Factors such as sex, age, education, and CD4 + T cell counts correlated with survival.

The existing research into the effects of TCM treatment on long-term survival of AIDS patients might be biased toward the null without accounting for selective bias. In particular, there are no results from randomized, controlled trials; thus, the effect of TCM on the survival of HIV/AIDS patient is still uncertain.

## Counteracting the adverse side effects of antiviral drugs

Although cART can reduce the morbidity and mortality of AIDS, it inevitably has adverse effects. The main adverse effects of antiviral drugs are myelosuppression, liver dysfunction, digestive discomfort (including diarrhea, abdominal distension, and pain), anaphylactic reaction, dyslipidemia, and sleep disorders. Patients in developing countries have a limited choice of antivirus strategies. Antiretroviral treatment is lifelong, and therefore demands good treatment adherence. However, some patients cannot endure the treatment’s adverse effects or cannot continue with cART because of other serious diseases. It is therefore very important to find methods to reduce drug toxicity or increase tolerance of antiviral drug toxicity. Research and clinical observations indicate that TCM can alleviate or improve some adverse effects.

The main ingredients of Wendan granule include *Inula japonica*, *Pericarpium citri reticulatae*, *Pinellia ternata*, *Atractylodes macrocephala*, *Semen raphani*, *Radix codonopsis*, and *Coptis chinensis*. This preparation regulates stomach qi and reduces phlegm, clears heat and dries damp, and eliminates vomiting and warms the stomach. A randomized, placebo-controlled trial investigated the effect of Wendan granule on alleviating gastrointestinal symptoms caused by antiretroviral therapy in 100 patients [27]. The incidence of gastrointestinal adverse symptoms caused by cART in the treatment group was 20.4 %, which was significantly lower than the incidence of 48.0 % in the placebo group. This study demonstrated that Wendan granule can reduce the incidence of gastrointestinal adverse symptoms caused by cART, and that TCM treatment can significantly reduce clinical symptoms, especially mild or moderate symptoms.

Jing yuan kang capsule is an herbal formulation composed of six Chinese herbs; the main ingredients are ginseng, *Rehmannia glutinosa*, *Angelica sinensis*, Herba *Epimedii*, *Astragalus membranaceus*, and *Spatholobus suberectus*. It tonifies qi, promotes blood circulation, strengthens the spleen, and reinforces the kidney. A randomized, double-blinded trial on 116 AIDS patients found that 6 weeks of systematic administration of Jing yuan kang capsule had a good therapeutic effect on peripheral leucopenia in the treatment group compared with the control group [28].

Danggui Shaoyao San is a famous Chinese herbal formula that nourishes blood, regulates the liver, nourishes the spleen, disinhibits dampness, and nourishes qi. Its main ingredients are *Angelica sinensis*, Radix *Paeoniae alba*, *Atractylodes macrocephala*, *Poria cocos*, *Alisma orientale,* Radix *Curcumae*, and *Hedyotis diffusa*. In one cohort study, a decoction based on Danggui Shaoyao San was used to treat cART-induced liver dysfunction in 48 patients [29]. The results showed that the decoction could counteract cART-induced liver dysfunction evidenced by dampening of elevated liver enzyme levels, and could alleviate symptoms such as abdominal distension, diarrhea, poor appetite, and hypochondriac pain.

Although there is some evidence that TCM can mitigate the adverse effects of cART, research in this field is still preliminary.

## Promoting immune reconstitution

HIV infection usually results in immunodeficiency. Immune reconstitution (IR) is observed when the immune response recovers to normal or near normal levels after cART or other treatments. This reduces the incidence of various AIDS-related opportunistic infections and tumors and reduces mortality and morbidity in AIDS patients. IR evaluation indexes include the number and functional recovery of CD4 + T cells, the stability of the immune activation state, and the restoration of lymph node structure. In addition to cART, adoptive immune cell therapy, bone marrow transplantation, and cytokine infusion can benefit IR [30]. However, not all patients can receive cART or experience full IR.

The holistic framework of TCM, its use of syndrome differentiation, and rich lore of herbal medicine treatment, mean that it is uniquely placed to promote IR. According to TCM theory, the essence of clinical manifestation is a deficiency of vital qi at the middle-late stage of AIDS. Therefore, strengthening qi is a fundamental TCM treatment therapy. Some research has demonstrated the important role of TCM in IR.

Immune No.2 is a Chinese hospital herbal preparation that includes Radix *Panacis quinquefolii*, *Astragalus membranaceus*, and *Viola yedoensis*. It tonifies qi, nourishes yin, and invigorates primordial qi. In a randomized, double-blinded, placebo-controlled clinical trial, Immune No.2 was used to promote IR in 233 patients with HIV/AIDS after cART [31]. The study showed that Immune No.2 can effectively improve the numbers of CD4 + T cells, CD45RA and CD45RO antigen counts, and the main clinical symptoms and signs of patients after cART, thereby promoting IR.

## Improvement of laboratory results

The two main goals of AIDS treatment are to eliminate viral loads and to raise CD4 + T cell counts. These two indexes have also been adopted in TCM treatment of AIDS, and a few studies have demonstrated that TCM is very promising in reducing HIV viral loads, increasing CD4 + T cell counts, or affecting other immunological indicators. Compared with the index of HIV viral loads, the CD4 + T cell count is currently the more popular measure of efficacy in TCM clinical trials for AIDS treatment.

Zhongyan-4 is a Chinese hospital herbal preparation that includes ginseng, *Fructus lycii*, Radix *Trichosanthis*, *Viola yedoensis*, and *Salvia miltiorrhiza*. It supplements qi, nourishes yin, clears heat, and detoxifies. In a randomized, double-blinded, parallel, placebo-controlled clinical trial, Zhongyan-4 was tested on 72 HIV/AIDS patients in the early or middle stage of the disease [32]. The findings suggested that the efficacy of Zhongyan-4 was superior to that of a placebo in elevating CD4 + T, CD45RA+, and CD8 + T counts, reducing HIV virus load, improving clinical symptoms or signs, and increasing body weight. No obvious adverse reactions were found.

Fuzheng Paidu granule consists of *Astragalus membranaceus*, ginseng, roasted Rhizoma *Atractylodis macrocephalae*, *Poria cocos*, Pericarpium *Citri reticulatae*, Fructus *Amomi*, *Semen coicis*, Radix *Morindae officinalis*, Herba *Cistanche deserticola*, Herba *Epimedii*, *Forsythia suspensa*, *Angelica sinensis*, and Radix *et Rhizoma glycyrrhizae*. It tonifies qi, nourishes yin, invigorates the spleen, clears heat, and detoxifies. A 6-month cohort study of 34 outpatients with HIV/AIDS tested the granule’s effects on regulation of immune activation molecules CD38 and human leukocyte antigen-D related to CD4 + T and CD8 + T cells [33]. The results showed that HIV/AIDS patients exhibited an immune activation profile following treatment. One potential mechanism of action for Fuzheng Paidu granule may lie in its ability to up-regulate CD38 and HLA-DR receptor levels on CD4 + T cells, and down-regulate them on CD8 + T cells, thereby modulating immune activation of CD4 + T and CD8 + T cells.

## Treatment safety

The combined use of herbal medicines and chemical medicines is common in clinical practice, since this kind of combination can not only improve treatment efficacy but also help to reduce drug dosages and adverse reactions. Herbal medicines and their effective ingredients probably induce or inhibit the action of drug metabolic enzymes and cause drug interactions. Therefore, the interaction between antiviral medicines and herbal medicines is an issue of concern.

Research has demonstrated that some herbal medicines may interact with antiviral drugs [34], which could interfere with antiviral drug metabolism and change the antiviral drug concentration [35]. However, clinical reports show that TCM is a relatively safe treatment method for HIV/AIDS, with only a few studies reporting minor side effects. There is no clinical research focusing on the interaction between TCM and antiviral drugs. Therefore, we suggest that future research on the simultaneous use of TCM and cART should pay more attention to antiviral drug metabolism.

## Conclusion

Although the discovery of cART has changed the prognosis of HIV-infected patients, and those treated with cART have a life expectancy similar to that of uninfected subjects, TCM has been used worldwide to treat AIDS and its complications.

This review revealed some new aspects of TCM clinical research on AIDS: use of the distinctive features of TCM to meet clinical demands, a focus on clinical signs and symptoms, and the use of different research designs that are methodologically robust. Research has also examined the pathogenesis, mechanism, efficacy, and safety of TCM. We believe that additional research will help to reveal the importance of TCM for treatment of AIDS.

## Additional file

Additional file 1:
**Multilingual abstracts in the six official working languages of the United Nations. **(PDF 228 kb)

## Abbreviations

AIDS: Acquired immune deficiency syndrome
HIV: Human immunodeficiency virus
CAM: Complementary and alternative medicine
TCM: Traditional Chinese medicine
WM: Western medicine
WHO: World Health Organization
CMPs: Chinese medicine professionals
cART: Combination antiretroviral therapy
QOL: Quality of life
IR: Immune reconstitution
