# Supplementary material for: Effectiveness and safety of traditional Chinese medicine in treating acquired immune deficiency syndrome: 2004–2014
Source: Infect Dis Poverty. 2015 Dec 23;4:59. doi: 10.1186/s40249-015-0093-6 (PMC4690280; doi:10.1186/s40249-015-0093-6)
Supplement: Additional file 1: — Multilingual abstracts in the six official working languages of the United Nations. (PDF 228 kb) [file 40249_2015_93_MOESM1_ESM.pdf]

Translation of the abstract into the six official working languages of the United Nations

فعالية وسلامة الطب الصيني التقليدي في علاج متلازمة نقص المناعة: 2004-2014

تشي-بن ليو، جي-بينج يانج، لي-ران شو

الخلاصة

تم إحراز تقدم ملحوظ في الصين في استخدام الطب الصيني التقليدي (TCM) لعلاج متلازمة نقص المناعة المكتسب (الإيدز). كان هدفنا استعراض أحدث التطورات في علاج الطب الصيني التقليدي للإيدز في الصين بين عامي 2004 و 2014. قمنا باستعراض محتوى المقالات الأصلية التي تحقق في فعالية وسلامة الطب الصيني التقليدي لعلاج الإيدز المنشورة في المجلات باللغتين الصينية والإنجليزية. تم العثور على المراجع ذات الصلة من 2004-2014 باستخدام بوب ميد وقاعدة بيانات معارف البنية التحتية الوطنية الصينية. ووجدنا أن الطب الصيني التقليدي قد استخدم على نطاق واسع لعلاج الإيدز ومضاعفاته في الصين. وقد ازداد عدد الدراسات عن الطب الصيني التقليدي مما يدل على فعاليته وسلامته. وشمل معايير الفعالية في المقالات المتناولة التخفيف من حدة فيروس نقص المناعة البشرية (HIV)-العلامات والأعراض ذات الصلة، وتحسين نوعية الحياة، وتحسين مدى البقاء على قيد الحياة، ومقاومة الآثار الجانبية الضارة للأدوية المضادة للفيروسات. وتقوية إعادة بناء المناعة، وتحسين النتائج المعملية. وخلاصة القول، أن المطبوعات تشير إلى أن الطب الصيني التقليدي آمن. وأن الطب الصيني التقليدي يلعب دوراً هاماً في علاج الإيدز. وقد حاولت بعض الدراسات قياس فعالية وسلامة الطب الصيني التقليدي لعلاج الإيدز، ولكن تحتاج إلى المزيد من الأدلة. لذلك، هناك حاجة إلى المزيد من البحث حول هذا الموضوع في المستقبل.

Translated from English version into Arabic by Saher Salama, through

TRANSLATORS
WITHOUT BORDERS

中医药治疗艾滋病的有效性和安全性：2004-2014

刘志斌，杨冀平，徐立然

摘要

在中国，中医药治疗艾滋病已经取得实质性进展；本文将回顾 2004 到 2014 年之间中医药治疗艾滋病取得的最新进展。我们述评了中医药治疗艾滋病有效性和安全性的中英文文献，相关文献来自于 PubMed 及 CNKI 数据库收录的 2004 年至 2014 年公开发表的论文。我们发现在中国中医药已经被广泛的用于艾滋病及其并发症的治疗。这些研究结果显示了中医药的有效性及安全性。中医的有效性主要有减轻 HIV 相关的症状和体征，增加患者生活质量，提高患者长期生存时间，减少抗病毒药物的副作用，促进免疫重建和改善实验室检查结果等。总之，文献表明中医药在治疗艾滋病及其并发症中有着重要作用，也是安全的。一些研究已经尝试评价中医药治疗艾滋病的有效性和安全性，但还需要更多的证据来支持。所以，依旧需要更多的研究来评价这一主题。

Translated from English version into Chinese by Liu Zhi-bin

L'efficacité et la fiabilité de la médecine traditionnelle chinoise dans le traitement du syndrome d'immunodéficience acquise : 2004-2014

Zhi-Bin Liu，Ji-Ping Yang，Li-Ran Xu

Résumé

Des progrès notables ont été réalisés en Chine dans l'utilisation de la médecine traditionnelle chinoise (MTC) pour le traitement du syndrome d'immunodéficience acquise (SIDA). Notre objectif était d'analyser les récents développements dans le traitement du SIDA en Chine par la MTC, entre 2004 et 2014. Nous avons analysé le contenu d'articles originaux enquêtant sur l'efficacité et la fiabilité de la MTC dans le traitement du SIDA, publiés dans des revues de langues chinoise et anglaise. Les références pertinentes pour les années 2004 à 2014 ont été trouvées grâce à PubMed ainsi qu'à la Base de Données de l'Infrastructure Nationale de la Connaissance de la

Chine. Nous avons constaté qu'en Chine, la MTC a été largement utilisée pour traiter le SIDA et ses complications. Le nombre d'études sur la MTC a augmenté, ce qui montre son efficacité et sa fiabilité. Les mesures de l'efficacité figurant dans les articles analysés ont inclus l'atténuation des signes et symptômes liés au virus d'immunodéficience humaine (VIH), des améliorations dans la qualité de vie ainsi que dans la survie sur le long terme, la neutralisation des effets indésirables des médicaments antiviraux, la promotion de la reconstitution immunitaire et l'amélioration des résultats de laboratoire. En résumé, ces documents indiquent que la MTC est fiable. La MTC joue un rôle important dans le traitement du SIDA. Quelques études ont tenté de mesurer l'efficacité et la fiabilité de la MTC dans le traitement du SIDA, mais d'autres preuves sont encore nécessaires. Par conséquent, il est nécessaire de réaliser de nouvelles recherches sur ce sujet dans le futur.

Translated from English version into French by paris9, through

Эффективность и безопасность традиционной китайской медицины в лечении синдрома приобретенного иммунодефицита, 2004–2014гг.

Zhi-Bin Liu，Ji-Ping Yang，Li-Ran Xu

Аннотация

В Китае был достигнут существенный прогресс в лечении синдрома приобретенного иммунодефицита (СПИД) благодаря использованию методов традиционной китайской медицины (ТКМ). Наша цель заключается в обзоре последних достижений ТКМ в лечении СПИДа в Китае в период с 2004 г. по 2014 г. Мы рассмотрели содержание оригинальных журнальных статей, исследующих эффективность и безопасность методов ТКМ в лечении СПИДа, опубликованных на китайском и английском языках. Соответствующие ссылки с 2004 г. по 2014 г. были найдены с помощью поисковой системы PubMed и китайской национальной инфраструктурной базы знаний. Мы обнаружили, что в Китае широко применяются методы ТКМ для лечения СПИДа и его осложнений. Количество исследований в области ТКМ возросло, что указывает на эффективность и безопасность ее методов лечения. Критерии эффективности в рассматриваемых статьях — это, помимо прочего, облегчение клинических проявлений и симптомов, связанных с вирусом иммунодефицита человека (ВИЧ), улучшение качества жизни, улучшение показателей долгосрочной выживаемости, нейтрализация побочных эффектов противовирусных препаратов, улучшение показателей иммуновосстановительной терапии и улучшение результатов лабораторных исследований. В целом, изучение литературы показывает, что ТКМ является безопасной. Кроме того, ТКМ играет важную роль в лечении СПИДа. В некоторых исследованиях предпринимались попытки измерить эффективность и безопасность ТКМ в лечении СПИДа, однако доказательств пока не достаточно. Таким образом, требуются дальнейшие исследованияв этой области.

Translated from English version into Russian by Anna Romanenko, through

Eficacia y seguridad de la medicina tradicional china en el tratamiento del síndrome de inmunodeficiencia adquirida: 2004-2014

Zhi-Bin Liu，Ji-Ping Yang，Li-Ran Xu

Resumen

En China Se ha hecho un progreso significativo en el uso de la medicina tradicional china (MTC) para el tratamiento del síndrome de inmunodeficiencia adquirida (SIDA). Nuestro objetivo fue analizar los últimos desarrollos en el tratamiento del SIDA con MTC en China entre los años 2004 y 2014. Analizamos el contenido de artículos originales que investigaban la eficacia y seguridad de la MTC para el tratamiento del SIDA publicados en revistas en chino y en inglés. Mediante el uso de PubMed y la Base de datos de la infraestructura de conocimiento nacional de China se encontraron referencias de relevancia entre el año 2004 y el año 2014. Encontramos que la MTC ha sido ampliamente utilizada para el tratamiento del SIDA y sus complicaciones en China. La cantidad de estudios MTC se ha incrementado, lo que indica su eficacia y seguridad. Las medidas de eficacia en los artículos analizados incluyeron el alivio de signos y síntomas asociados al virus de la inmunodeficiencia humana (VIH), mejoras en la calidad de vida, mejoras en la supervivencia a largo plazo, neutralización de los efectos secundarios adversos de los medicamentos antivirales, promoción de la reconstitución del sistema inmune y mejora de los resultados de laboratorio. En suma, la literatura indica que la MTC es segura. La MTC juega un papel importante en el tratamiento del SIDA. Algunos estudios han intentado medir la eficacia y seguridad de la MTC para el tratamiento del SIDA, pero se necesita mayor evidencia. Por lo tanto, en el futuro se necesita de mayor investigación sobre este tema.

Translated from English version into Spanish by Maria Alejandra Aguada, through

TRANSLATORS
WITHOUT BORDERS
